# Efficacy and Safety of Anthelmintics Tested against *Taenia solium* Cysticercosis in Pigs

**DOI:** 10.1371/journal.pntd.0002200

**Published:** 2013-07-25

**Authors:** Ernatus Martin Mkupasi, Chummy Sikalizyo Sikasunge, Helena Aminiel Ngowi, Maria Vang Johansen

**Affiliations:** 1 Department of Veterinary Disease Biology, Faculty of Health and Medical Sciences, University of Copenhagen, Frederiksberg, Denmark; 2 Department of Para-clinical Studies, School of Veterinary Medicine, University of Zambia, Lusaka, Zambia; 3 Department of Veterinary Medicine and Public Health, Sokoine University of Agriculture, Morogoro, Tanzania; Universidad Peruana Cayetano Heredia, Peru

## Abstract

Porcine cysticercosis, an infection caused by *Taenia solium* metacestodes, is continuously being reported in low-income countries of Latin America, Asia, and sub-Saharan Africa. The disease was declared eradicable by the International Task Force for Diseases Eradication (ITFDE) in 1993, and it is listed among the 17 WHO Neglected Tropical Diseases and Neglected Zoonoses that are potentially eradicable. In view of that, WHO has proposed a step-wise approach to its elimination, including chemotherapy of infected pigs. Different drugs have been tested on porcine cysticercosis with varying efficacies. These include flubendazole, fenbendazole, albendazole, albendazole sulphoxide, oxfendazole, praziquantel, and nitazoxanide. This review summarises available information on the efficacies and adverse effects shown by these drugs in pigs. Oxfendazole has shown to be effective for the control of porcine cysticercosis; however, it needs to be integrated with other control approaches. There is a need for standardised guidelines for evaluating the efficacy of anthelmintics against porcine cysticercosis, and more efficacy studies are needed since the conclusions so far are based on a limited number of studies using few infected pigs.

**Key Learning Points**

- Among the anthelmintics evaluated, oxfendazole has proven to be safe and efficacious against porcine cysticercosis.
- Limited intervention trials have shown oxfendazole to be effective for control of porcine cysticercosis; however, it needs to be integrated with other control approaches.
- Standardised protocols for assessment of anthelmintic efficacy and effectiveness for control of porcine cysticercosis are lacking, making inter-study comparison difficult.
- More studies are needed to fully assess the efficacy and effectiveness of oxfendazole and other anthelmintics against *T. solium* cysticercosis and other pig parasitoses.

**Key References**

- Gonzales AE, Garcia HH, Gilman RH, Gavidia CM, Tsang VCW, et al. (1996) Effective, single-dose treatment of porcine cysticercosis with oxfendazole. Am J Trop Med Hyg 54: 391-394.
- Gonzalez AE, Falcon N, Gavidia C, Garcia HH, Tsang VCW, et al. (1997) Treatment of porcine cysticercosis with oxfendazole: a dose-response trial. Vet Rec 141: 420-422.
- Moreno L, Lopez-Urbina MT, Faras C, Domingue G, Donadeu M, et al. (2012) A high oxfendazole dose to control porcine cysticercosis: Pharmacokinetics and tissue residue profiles. Food Chem Toxicol 50: 3819 – 3825.
- Sikasunge CS, Johansen MV, Willingham AL, III, Leifsson PS, Phiri IK (2008) Taenia solium porcine cysticercosis: viability of cysticerci and persistency of antibodies and cysticercal antigens after treatment with oxfendazole. Vet Parasitol 158: 57-66.
- Torres A, Plancarte A, Villalobos ANM, Aluja ASd, Navarro R, et al. (1992) Praziquantel treatment of porcine brain and muscle Taenia solium cysticercosis. 3. Effect of 1-day treatment. Parasitol Res 78: 161-164.

## Introduction

Making safe and evidence-based recommendations for porcine cysticercosis treatment requires adequate information on the efficacy and safety of the anthelmintics to be used [1]. It has been reported that the policy of confiscating cysticercosis-infected pig carcasses from official slaughter houses without compensation only drives farmers to illegal slaughter and consumption or sale, increasing the possibility of infecting more people [2]. Effective treatment of infected pigs will increase the value of pork and access to formal marketing systems, which will be strong incentives for farmers to treat their pigs and increase compliance with other control activities such as health education, sanitation, and human treatment. However, in order to improve the economic value of pork, its appearance should be clean and similar to noninfected meat [3].

Efforts to treat *T. solium* cysticercosis in pigs started in late 70s after promising results from human neurocysticercosis treatment with praziquantel. Since then, seven anthelmintics have been evaluated in treatment of porcine cysticercosis employing naturally infected pigs [4]. The seven anthelmintics comprise five benzimidazoles (BZ), praziquantel, and nitazoxanide. The BZ represents a class of broad-spectrum anthelmintics, which have the great merit of being available in a wide variety of formulations and routes of administration [5]. Since their introduction in the early 60s, they have had a major impact on safe and effective management of helminths in both humans and animals [6], [7]. Praziquantel, on the other hand, is a prazinoisoquinoline derivative, and is highly effective against a broad range of trematodes and cestodes [8]. Nitazoxanide is a nitrothiazolyl-salicylamide derivative, the prodrug which is metabolised to form tizoxanide, the main active metabolite [9]. This work focuses on evaluating the efficacy and safety of flubendazole, fenbendazole, albendazole, albendazole sulphoxide, oxfendazole, praziquantel, and nitazoxanide anthelmintics, which have been tested for treatment of porcine cysticercosis.

A literature search was performed using the databases CAB, Web of Science, Medline, Agricola, and Agris, provided by the University of Copenhagen Library. Porcine cysticercosis treatment information published in English through August 2012 was evaluated. A total of twelve publications about porcine cysticercosis chemotherapy were obtained and summarised in this review. As no formal guidelines exist for assessing drug efficacy against porcine cysticercosis, the studies vary significantly regarding setup, number of pigs included, methodology, and ways of assessing and reporting the outcome [10]. For each study, we have included information about the dependent and independent variables, methodology, and duration of the study. The summary of the drugs' efficacy and safety are presented in the Table 1 and Table 2, respectively.

**Table 1:** Efficacy of different anthelmintics tested against porcine cysticercosis.

Reference	Anthelmintic	Dose (mg/kg)	Treated pigs	Control pigs	TE (wks)	KR	Effect on MC	Effect on BC
12	FLU	5.5–40	15	11	1–4	0–100	Variable	Variable
14	FBZ	5–45	16	2	3–5	NR	Good	Variable
17	ABZ	30 & 50	12	5	10–12	89–99	Good	Variable
20	ABZSO	15	4	3	12	59–100	Variable	Variable
23	PZQ	50	4	4	10–12	0–10	No effect	No effect
23	PZQ+OFZ	30+50	4	,,	10–12	100	Good	Good
23	OFZ	30	4	,,	10–12	100	Good	Good
24	OFZ	10	6	6	10	88–96	Good	Low
24	OFZ	20	6	,,	10	100	Good	Good
24	OFZ	30	5	,,	10	100	Good	Good
25	OFZ	30	16	4	1–12	80–100	Good	Variable
26	OFZ	30	20	40	32	100	Good	NE
27	OFZ	30	20	20	1–26	82–100	Good	Low
32	PZQ	50	11	2	1–65	20–75	Variable	Variable
33	PZQ	25	6	6	5	55–100	Variable	Variable
33	PZQ	50	6	,,	5	78–100	Good	Variable
33	PZQ	100	6	,,	5	18–100	Variable	Variable
34	NZX	150	6	6	10	0	No effect	No effect

KR - Killing rate, TE - Time evaluated, MC - Muscular cysts, BC - Brain cysts, NE - Not evaluated, NR - Not reported, wks - weeks.

**Table 2:** Reported adverse effects after anthelmintics treatment of porcine cysticercosis.

Reference	Anthelmintic	Dose (mg/kg)	Treatment duration	Adverse effects
12	FLU	5.5–40	10 consecutive days	Reduced appetite
14	FBZ	5–45	7 or 14 consecutive days	Slightly reduced appetite
17	ABZ	30	3 consecutive days	Lethargy and anorexia
17	ABZ	50	Single-dose treatment	Prostration, anorexia, lethargia, and death
20	ABZSO	15	8 consecutive days	Ulcers developed in 2 pigs at injection site
23	PZQ	50	Single dose	No side effect reported
23	PZQ+OFZ	30+50	Single dose	No side effect reported
23	OFZ	30	Single dose	No side effect reported
24	OFZ	10, 20 & 30	Single dose	No side effect observed
25	OFZ	30	Single dose	No side effect observed
26	OFZ	30	Single dose	Not reported
27	OFZ	30	Single dose	Not reported
32	PZQ	50	>15 days	Not reported
33	PZQ	25, 50 & 100	Single day	Not reported
34	NZX	150	7 consecutive days	No side effect observed

## Flubendazole (FLU)

Flubendazole has both antinematodal and anticestodal activities [11]. The drug is registered for use in pigs for control of helminth infections other than *T. solium* cysticercosis with doses between 1.6–4 mg/kg [5]. Early efforts to control porcine cysticercosis evaluated the effect of FLU in two treatment trials. In the first experiment conducted in Mexico [12], the efficacy of FLU on *T. solium* metacestodes was determined using 17 naturally infected pigs. Nine pigs were allocated into two groups of five and four pigs and treated with individual doses ranging from 5.5–6.4 mg/kg and 8.3–28.5 mg/kg, respectively. The drug was administered orally mixed in feed twice a day for ten consecutive days, while eight pigs were left untreated (Table 1).

The efficacy of the drug was evaluated 25 days after treatment by morphological changes, viability test, and histological assessment of the metacestodes in the treated pigs as compared to the control. In treated pigs, the metacestodes showed degenerative changes, being smaller, dry, and yellowish in color. The doses between 5.5–6.4 mg/kg showed low efficacy, as these had 82–98% of the metacestodes viable, while doses between 8.3–28.5 mg/kg had only 0–8% metacestodes viable, as compared to control pigs in which 60–100% metacestodes were viable. The study did not differentiate the effect of the treatment according to the location of metacestodes either in muscles or in brain tissues (Table 1).

In the second experiment [12], nine naturally infected pigs were divided into treatment and control groups comprising six and three pigs, respectively. The treated pigs were administered FLU mixed in feed in a dosage of 40 mg/kg daily for ten consecutive days. One pig died from an undetermined cause on day seven and was evaluated for the effect of treatment. Two pigs were sacrificed on days 12 and 14, respectively and the remaining three pigs on day 25. Three percent of muscular metacestodes were viable in the pig evaluated on day seven; the remainder had no viable metacestodes with exception of one pig evaluated on day 14, which had almost 100% viable muscular metacestodes. However, the pig was reported to have been reluctant to eat the medicated feed. The viability of brain metacestodes was found to be 28% in the pig evaluated on day seven and 98% in the pig evaluated on day 14. In the control group, the viability was about 98 and 88% for muscular and brain metacestodes, respectively (Table 1).

Histopathological examination further revealed destruction of metacestodes, which appeared swollen, edematous with acute inflammatory reaction seven days after treatment. At day 25, the metacestodes were completely destroyed with tissue necrosis. No adverse effects were reported at the dosage used apart from one pig from the second study, which died on day seven of treatment (Table 2). Doses between 8.3–40 mg/kg were found to be effective against metacestodes in muscles, but were less effective for brain metacestodes. The repeated treatment required makes it uneconomical and impractical for porcine cysticercosis control under field conditions in endemic areas.

## Fenbendazole (FBZ)

Fenbendazole has a broad spectrum of activity against gastrointestinal and respiratory helminths in domestic animals. It is registered for use in pigs at a dose of 5 mg/kg [13]. The efficacy of FBZ in treatment of porcine cysticercosis was evaluated in Korea [14], using 18 naturally infected pigs. The pigs were allocated into nine groups of two pigs each; eight groups were treated with a pig feed formulation of the drug and one group served as an untreated control. The first treated group was administered 45 mg/kg and the second group 20 mg/kg FBZ for 14 consecutive days. The third and fourth groups were both given 25 mg/kg and the fifth and sixth groups received 15 mg/kg also for 14 consecutive days. The remaining two groups were treated with 5 mg/kg per day for seven consecutive days.

The efficacy of the drug was assessed by sacrificing the pigs at days 20–39 after last treatment, and the metacestode morphological changes were evaluated grossly and by use of scanning and transmission electron microscopes. The study showed that FBZ was effective at all doses and regimens used (Table 1). The metacestodes in muscle were more affected than in the brain; they were deflated and shrunk and appeared as small rice grains in size and shape. However, the effect on the brain metacestodes varied as the cysts appeared to be in the process of disintegration. Generally FBZ was found to be safe; the only reported side effect was the slight decrease in appetite during the treatment in some treated pigs, but eventually they gained weight as compared to the controls (Table 2). Based on the treatment of four pigs, it was concluded that FBZ at 5 mg/kg for seven consecutive days was effective in treatment of porcine cysticercosis. However, the prolonged treatment duration required limits its use in control of porcine cysticercosis in endemic areas.

## Albendazole (ABZ)

Albendazole, another BZ anthelmintic, is oxidized to albendazole sulphoxide, the systematically active metabolite, and then further oxidized to albendazole sulphone, which is inactive [15]. It is a broad-spectrum anthelmintic that is effective against nematode, trematode, and cestode infections in sheep and cattle at a dose between 5–10 mg/kg [6], [15]. It has been reported to have better efficacy in treatment of human neurocysticercosis than praziquantel [16]. It is not registered for use in pigs.

An evaluation of the efficacy of ABZ in single- and multiple-dose regimens in treatment of porcine cysticercosis in naturally infected pigs was conducted [17]. Seventeen pigs were allocated into two treatment groups of six pigs each and a control group of five pigs. The first treatment group was administered with ABZ in a single dose of 50 mg/kg, the second group with 30 mg/kg every day for three days given orally mixed in feed, and the third control group was left untreated (Table 1).

The efficacy was assessed 10–12 weeks after treatment; the pigs were sacrificed and the effect of the treatment evaluated by metacestodes count and viability test. The three-day regimen of 30 mg/kg was effective, killing all metacestodes in muscles but not the brain metacestodes. In this group, the pigs developed adverse effects such as lethargy and anorexia (Table 2). In the group treated with a single dose of 50 mg/kg, the drug was less effective compared to 30 mg/kg for three days, leaving 11.4% and 6.7% viable metacestodes in muscular and brain tissues, respectively (Table 1). In addition, the pigs developed marked adverse effects such as severe prostration, complete anorexia, and lethargy, and one pig died on the third day after treatment (Table 2). In both groups, the calcified metacestodes were still visible in meat 10–12 weeks after treatment. The authors concluded, based on the 12 treated pigs, that although the three-day administration of albendazole was highly effective, the side effects observed and the multiple doses limit its applicability in the field. Additionally, the prolonged use of the drug in humans [18] and in dogs and cats [19] is reported to induce bone marrow toxicosis, which may lead to lethal pancytopenia.

## Albendazole sulphoxide (ABZSO)

Albendazole sulphoxide is available for use in ruminants for control of intestinal nematodes and some trematodes and cestodes at doses between 5–7.5 mg/kg [6]. It is not registered for use in pigs. The efficacy of ABZSO against porcine cysticercosis was evaluated in Mexico [20]. Seven naturally infected pigs (four sows and two boars) with age ranging between 6–12 months were divided into two groups of four and three pigs for treatment and control, respectively. The treatment group received a dosage of 15 mg/kg ABZSO by subcutaneous injection for eight consecutive days, and the control group was given a 0.9% saline solution (Table 1).

The efficacy of ABZSO was reported to be 100% in the muscle metacestodes when evaluated 12 weeks after treatment. The metacestodes were examined macroscopically for vesicular or micro-calcified cysts and by viability test. The metacestodes were reported to be degenerated but were still visible 12 weeks post treatment. ABZSO was ineffective against brain metacestodes as it left 41.1% of the metacestodes viable. In the control group, 91.5% of muscular and 75.8% of brain metacestodes were viable. Histopathological evaluation of muscle tissues indicated complete destruction of the metacestode with necrosis. The treatment was reported to be well tolerated; however, two pigs developed ulcers at injection sites. Based on the treatment of four pigs, it was concluded that 15 mg/kg ABZSO was 100% effective for muscle cysts treatment but less effective for brain cysts. However, the required multiple treatments and low efficacy to the brain cysts limits its use in control of porcine cysticercosis.

## Oxfendazole (OFZ)

Oxfendazole has broad-spectrum activity against inhibited larval stages of gastrointestinal roundworms, tapeworms, and lungworms in many animal species at doses between 4.5–10 mg/kg [21]. Unlike other BZ compounds, OFZ is absorbed more readily from the gastrointestinal tract. OFZ is metabolically interconvertable to FBZ, and then extensively metabolised to fenbendazole sulphoxide, an active metabolite, then oxidized to the inactive fenbendazole sulphone. This is responsible for prolonged systemic bioavailability of the drug, accounting for the reported high efficacy against porcine cysticercosis. Following oral administration at 30 mg/kg in pigs, it rapidly absorbed, reaching very high plasma levels and systemic exposure after two hours, and persisted in plasma up to 96 hours post treatment. The elimination half-life is about 7.5 hours in sheep. The dose is well tolerated in all tested pigs [21], [22]. However, the drug is not registered for use in pigs.

Studies have been conducted to evaluate OFZ's efficacy, safety, killing time, and cyst clearance rates in pigs naturally infected with porcine cysticercosis (Table 1). In the first study conducted in Peru [23], sixteen adult pigs naturally infected with *T. solium* cysticercosis were divided into four groups that were randomly allocated to PZQ, PZQ plus OFZ, and OFZ treatment groups and a control group, respectively. The pigs were kept at a veterinary facility, free from infection transmission throughout the study period. The drugs were mixed in feed and administered orally. In the groups treated with OFZ alone or the PZQ plus OFZ combination, the drugs were found to be 100% effective as no metacestodes evaginated from either muscle or brain tissue 10–12 weeks after treatment, as compared to controls where nearly 100% viable metacestodes were found (Table 1). Praziquantel at 50 mg/kg alone was not effective as nearly 100% of metacestodes were viable in both muscle and brain tissue, similar to the control group [23]. The dosage regime was safe as no side effect were noted during the observation period.

In another study [24], 24 adult pigs naturally infected with cysticercosis were allocated into four groups and treated with 10, 20, and 30 mg/kg OFZ mixed with feed or left untreated, respectively. The pigs were kept at the veterinary facility, free from any risk of the transmission of cysticercosis, for the whole study period. The pigs were sacrificed eight to ten weeks post treatment for metacestodes evaluation. Up to 25 metacestodes from selected muscles, heart, and brain were excised and tested for viability by evagination. Doses of 10 and 20 mg/kg were not effective as 4–12% of metacestodes were still viable in both muscle and brain tissues, but 30 mg/kg OFZ showed high efficacy as no metacestodes were viable in either muscle or brain tissue. More than 75% of the cysts in the control group were viable irrespective of the anatomical location (Table 1).

The time taken for OFZ to kill the cysticerci was evaluated using 20 adult pigs naturally infected with cysticercosis, where 16 pigs were treated with 30 mg/kg OFZ and four were left untreated [25]. The cysticercosis-infected pigs were brought to and reared at an infection-free veterinary facility for the duration of the study. The OFZ-treated pigs were sacrificed at weeks one, four, and 12 after treatment, where all control pigs were sacrificed at week 12. The viability of metacestodes was assessed by evagination test. It was observed that the metacestode viability was significantly reduced one week following OFZ treatment, but at week four some viable metacestodes were still observed. At week twelve, all metacestodes had disappeared leaving only minuscule scars except in one animal that had viable brain metacestodes. In the controls, the average metacestode viability was 66% and 58% from muscle and brain tissue, respectively.

Protection against reinfection after OFZ treatment was evaluated using 20 adult pigs naturally infected with cysticercosis [26]. The pigs were drenched with 30 mg/kg OFZ and kept in the disease-free facility in the veterinary faculty for eight weeks. After that period, each treated pig was matched with two naïve pigs similar in age and sex, obtained from disease-free farms, and exposed in a high-risk environment for the infection. Three months later, the pigs in both groups were dissected to evaluate their infection status. New infections were detected in 15 of the 32 controls (42%) by serology and by the presence of metacestodes in 12 pigs (37%), whereas no viable metacestodes were detected in the muscles of the previously treated pigs. From this study, the OFZ was demonstrated to have an added advantage: that following treatment, infected pigs were protected against reinfection for at least three months [26].

The efficacy, killing time, and cyst clearance rate in *T. solium*–infected pigs treated with OFZ was evaluated in Zambia using 40 (22 males and 18 females) infected pigs [27]. The pigs were kept in a disease-free facility at veterinary school premises. They were divided into treated and control groups; the treated group received 30 mg/kg OFZ given orally mixed in feed. The pigs were sacrificed at one, four, eight, and 26 weeks after treatment. In pigs killed at one week, both live and dead metacestodes were recovered with mean (± S.D.) of 18±17% and 92±5% in treated and control groups, respectively. However, one striking finding at one week post treatment was that in treated pigs all metacestodes in the heart were dead. At four and eight weeks after treatment, all muscle metacestodes were calcified, but brain metacestodes were viable. At 26 weeks post treatment, in four out of five carcasses the metacestodes had completely disappeared with normal meat appearance, but one carcass had visible calcified metacestodes in the muscles. The brain metacestodes were still viable with mean (± S.D.) of 36±49% and 91±8% in treated and control groups, respectively. From this study, it was concluded that OFZ at 30 mg/kg body weight killed all muscular metacestodes as early as four weeks after treatment, but brain metacestodes were not significantly affected. It was demonstrated that complete clearance of metacestodes occurred between 8–26 weeks or more. The authors hypothesized that the clearance of the dead metacestodes from the muscle tissue depends on the intensity of the infection at the time of treatment [27]. In three treatment trials [23]–[25], the dose of 30 mg/kg OFZ was reported to be safe in infected pigs (Table 2).

An intervention field treatment trial using OFZ in mass chemotherapy of pigs in endemic villages in Peru in combination with taeniasis chemotherapy in humans using PZQ as a control program has been reported with promising results [28]. A single dose of 5 mg of PZQ was given to eliminate intestinal taeniasis in humans, and two rounds of OFZ (30 mg/kg) were administered to all pigs. There was a decrease of prevalence of the condition at the end of the intervention period. The impact of the intervention was low and did not attain the goal of elimination of transmission due to less than 100% coverage in both human and pig populations.

In another study, a randomized controlled trial was conducted to evaluate the effectiveness of OFZ treatment for control of porcine cysticercosis in endemic villages of Mozambique [29]. A substantial benefit of treating pigs with OFZ using the single oral dose of 30 mg/kg was clearly demonstrated, since the prevalence and incidence in groups of treated pigs was significantly lower compared to the group of untreated pigs [29]. The results from the two intervention studies showed that OFZ treatment was effective to control porcine cysticercosis; however, it needs to be integrated with other control approaches.

In all studies, 30 mg/kg OFZ was 100% effective in treating muscle cysticerci with variable efficacies in the brain cysticerci. The reason for the reported low efficacy against the brain cysticerci is not clear; genetic variation of the cysticerci or differences in location of the cysticerci within the brain tissues may have influenced the effectiveness of OFZ treatment. However, in most endemic areas pork goes uninspected, but the pig brain is not commonly eaten raw; thus, it is not likely that cysts that survive only in the brain will be ingested and perpetuate the cycle. The withdrawal period has been recently reported to be 17 days, making it safe for pre-slaughter treatment of infected pigs [22]. The use of OFZ in pregnant sows at 13.5 mg/kg has been reported to be safe [30]; however, the 30 mg/kg dose recommended for *T. solium* cysticercosis treatment needs to be further evaluated. The safety of the dose to the piglets should also be evaluated. In spite of the mentioned limitations, the drug is safe, inexpensive, and easily administered in suspension preparation, making it a good candidate to be promoted for use in endemic areas.

Although OFZ worked significantly better than the other BZ, it is not ideal, as studies have shown variable effects on brain metacestodes. More research is needed to fully assess the efficacy and effectiveness of OFZ against *T. solium* cysticercosis and other pig parasitoses before it can be recommended for use as a regular anthelmintic in endemic areas. The drug needs to be registered for use in pigs and made available in cysticercosis endemic areas.

## Praziquantel (PZQ)

Praziquantel is effective against a wide range of trematodes and cestodes in dogs and cats at a dose of 5 mg/kg, being generally less effective against nematodes [8], [31]. It is not registered for use in pigs. The use of PZQ for control of porcine cysticercosis at different doses has been evaluated (Table 1). In the first experiment, the effect of PZQ treatment was evaluated using 13 naturally infected adult pigs, divided into two groups of 11 and two pigs [32]. The first group was treated with PZQ at 50 mg/kg per day, divided into three portions mixed in feed for a period of 15 days. The second group of two pigs was the infected control, left untreated and monitored for 13 months. Nine noninfected pigs were used as noninfected treated controls.

The efficacy of the treatment was monitored through CT scans and histopathogical and viability evaluations of the metacestodes. Results of the CT scans indicated both morphological and size change following treatment. Metacestodes became irregular, smaller, and less clearly defined after treatment. Although the decrease in number of metacestodes was observed in both brain and muscle tissues from the first day after treatment, some metacestodes were still visible on day 47 after the last treatment. For morphological and viability evaluation, samples were taken from the masseters of two pigs at days 1 and 8 after the first treatment and then from pigs killed on 2, 5, 7, 10, 15, 25, 28, 32, 57, 63, and 65 days after the last dose of PZQ. The two untreated pigs were necropsied after 13 months. Macroscopic evaluations of muscles showed sequential changes in metacestodes. At first, the vesicular fluid became turbid with white capsule of varying thickness. In more advanced stages, either the tissue cavities where the parasite lodged contained yellowish, caseous materials, or the parasites looked like small rice grains. From day 57 onward, muscle parasites could not be identified, only small scars suggesting their previous presence.

The results of the viability study indicated that only the pig evaluated on day one during treatment had 8/10 (80%) viable muscle metacestodes, while in those evaluated on the following days all muscle metacestodes were dead. Viable brain metacestodes were found in two treated pigs; one on day 15 (1/6) and the other on day 25 (2/8). Histopathologically, the metacestodes in muscles showed severe degenerative changes. In the brain, the results were variable as many metacestodes were intact, surrounded with limited inflammatory infiltrate. It was concluded that the drug damaged the metacestodes in muscles, as demonstrated by morphological change and inhibition of evagination afterward, and that the inflammatory reaction killed and eliminated the metacestodes.

In efforts to overcome the limitations of multiple doses of 50 mg/kg PZQ [32], a one-day PZQ treatment at three different doses of 25, 50, and 100 mg/kg against muscular and brain metacestodes was evaluated in Mexico [33]. Twenty-four naturally infected pigs divided into four groups of six animals each were employed. Three groups were administered with a total dose of 25, 50, and 100 mg/kg, respectively, given in three portions in the morning, afternoon, and evening mixed in feed. The fourth group was left untreated. The effect of the treatment was assessed through morphological, histological, and viability evaluations.

Thirty-four days post treatment, all pigs were sacrificed and cysts were evaluated. Macroscopic examination revealed viable cysts in all six untreated pigs, as compared to 4/6 in those treated with 25 mg/kg and 3/6 in the groups treated with 50 or 100 mg/kg. Metacestodes were degenerated, appearing as small rice grain in most pigs from the treated groups. Following viability evaluation in the group treated with 25 mg/kg, viable cysts were observed in 4/5 pigs with viability ranging between 5–45% and 15–30% for muscle and brain metacestodes, respectively. In the 50 mg/kg treated group, viable cysts were observed in 3/6 pigs with viability of 18–22% and 5–22% for muscle and brain metacestodes, respectively. In the 100 mg/kg treated group, a viability of 20–72% and 10–22% for muscle and brain mesacestodes was observed, respectively, in 3/6 pigs. While in the control group, the viability ranged between 68–100% and 70–100% in muscle and brain cysts, respectively (Table 1).

Most metacestodes in the muscles and brain of all treated pigs exhibited a histological picture of dying or dead parasites except in two pigs that were similar to the control group. The study showed that the treatment had some effect on the metacestodes, and that the effect correlated positively with increasing dose as evaluated by viability and histological statuses of the metacestodes at 34 days post treatment. A single dose of 50 mg/kg PZQ reduced the number of metacestodes but did not affect the viability of the remaining metacestodes. This study did not demonstrate the time taken to clear cysts from the meat as the pigs were evaluated one month after treatment.

Though good efficacy has been demonstrated using 50 mg/kg PZQ for 15 days in treatment of porcine cysticercosis [32], the long duration of treatment will be costly and impractical for the control of porcine cysticercosis in endemic areas. The safety of the treatment regimens for the infected pigs was not reported.

## Nitazoxanide (NZX)

A recent study was conducted in Peru using naturally infected pigs to explore the efficacy of combined therapy (ABZ plus PZQ) versus ABZ, PZQ, OFZ, or NZX alone at the dose of 15, 75, 30, and 150 mg/kg, respectively [34]. Fifty-four infected pigs were used, randomly allocated into nine groups with six pigs each. All drugs were administered orally, mixed with feed. The pigs were kept at the School of Veterinary Medicine facilities for ten weeks and then euthanized and subjected to necropsy study to determine the efficacy of the treatment.

Results showed that a combination of ABZ and PZQ, ABZ alone, and OFZ alone were effective antiparasitic drugs for treatment of cysticercosis, and the combined schemes had the best antiparasitic effect in the brain with a statistically significant reduction in viable brain cysts. Nitazoxanide at 150 mg/kg for seven consecutive days was not effective as it left many surviving cysts similar to control pigs, suggesting minimal or no cysticidal effect [34].

## Conclusion

The evaluated studies varied greatly in design and sample size, methodology was insufficiently described, and almost all studies reported different ways of assessing viability, a key dependent variable for evaluating efficacy. Time points for assessing efficacy, killing, or cyst clearance rates vary between the studies, making comparison between them difficult. Most publications inadequately recorded adverse drug reactions [35]. Due to difficulties in attaining an artificial infection pig model under laboratory conditions, recruiting homogenous groups as much as possible is suggested. Young infected pigs between 3–6 months of age are suggested to avoid the effect of natural involution of the parasite rather than the effect of the drug evaluated. The pigs should be randomized according to age, sex, and level of infection based on either number of tongue cysts and/or level of cysticercal antigens. Also, each group should not have less than six animals [10]. Based on the observations from the studies conducted and reviewed here, the cysts killing time and clearance rate should be evaluated at four and 12 weeks, respectively. Apart from ABZSO, all tested drugs were applied orally mixed in feed, which might be practical, but since BZ have generally poor absorption after oral administration, alternative routes of administration might be more appropriate when testing efficacy against a muscle and brain parasite.

Although highly diverse in setup and methodologies, so far OFZ has proven to be the most promising drug since a single dose of 30 mg/kg has been shown to have high efficacy against muscle metacestodes and is safe, but is less effective for brain metacestodes. This warrants further systematic assessment using alternative formulations and routes of administration. However, no standardised guidelines are in place for assessing the efficacy of anthelmintics against porcine cysticercosis, unlike most other pig helminths [10], and are urgently needed. In conclusion, although the WHO now recommends anthelmintic treatment of pigs as a way to control and eliminate *T. solium* cysticercosis, we still lack the tool to do so. Proper assessment of efficacy, effectiveness, registration of the drug in a suitable pig formulation, and availability of the drug in the endemic areas will be the way forward.
